# Animal and Organoid Models of Liver Fibrosis

**DOI:** 10.3389/fphys.2021.666138

**Published:** 2021-05-26

**Authors:** Yu-long Bao, Li Wang, Hai-ting Pan, Tai-ran Zhang, Ya-hong Chen, Shan-jing Xu, Xin-li Mao, Shao-wei Li

**Affiliations:** ^1^College of Basic Medicine, Inner Mongolia Medical University, Hohhot, China; ^2^Health Management Center, Taizhou Hospital of Zhejiang Province Affiliated to Wenzhou Medical University, Linhai, China; ^3^School of Medicine, Shaoxing University, Shaoxing, Chian; ^4^Key Laboratory of Minimally Invasive Techniques & Rapid Rehabilitation of Digestive System Tumor, Taizhou Hospital of Zhejiang Province Affiliated to Wenzhou Medical University, Linhai, China; ^5^Department of Gastroenterology, Taizhou Hospital of Zhejiang Province Affiliated to Wenzhou Medical University, Linhai, China

**Keywords:** liver, fibrosis, animal, organoid, model

## Abstract

Liver fibrosis refers to the process underlying the development of chronic liver diseases, wherein liver cells are repeatedly destroyed and regenerated, which leads to an excessive deposition and abnormal distribution of the extracellular matrix such as collagen, glycoprotein and proteoglycan in the liver. Liver fibrosis thus constitutes the pathological repair response of the liver to chronic injury. Hepatic fibrosis is a key step in the progression of chronic liver disease to cirrhosis and an important factor affecting the prognosis of chronic liver disease. Further development of liver fibrosis may lead to structural disorders of the liver, nodular regeneration of hepatocytes and the formation of cirrhosis. Hepatic fibrosis is histologically reversible if treated aggressively during this period, but when fibrosis progresses to the stage of cirrhosis, reversal is very difficult, resulting in a poor prognosis. There are many causes of liver fibrosis, including liver injury caused by drugs, viral hepatitis, alcoholic liver, fatty liver and autoimmune disease. The mechanism underlying hepatic fibrosis differs among etiologies. The establishment of an appropriate animal model of liver fibrosis is not only an important basis for the in-depth study of the pathogenesis of liver fibrosis but also an important means for clinical experts to select drugs for the prevention and treatment of liver fibrosis. The present study focused on the modeling methods and fibrosis characteristics of different animal models of liver fibrosis, such as a chemical-induced liver fibrosis model, autoimmune liver fibrosis model, cholestatic liver fibrosis model, alcoholic liver fibrosis model and non-alcoholic liver fibrosis model. In addition, we also summarize the research and application prospects concerning new organoids in liver fibrosis models proposed in recent years. A suitable animal model of liver fibrosis and organoid fibrosis model that closely resemble the physiological state of the human body will provide bases for the in-depth study of the pathogenesis of liver fibrosis and the development of therapeutic drugs.

## Introduction

Liver fibrosis is a pathophysiological process caused by a variety of pathogenic factors that induce the abnormal proliferation of connective tissue in the liver. The repair and healing processes of liver injury can be accompanied by the development of liver fibrosis, and if the factors underlying such injury are not addressed, the process of fibrosis continues, eventually leading to cirrhosis (Hernandez-Gea and Friedman, 2011; Parola and Pinzani, 2019). Viruses, toxins, drugs, alcohol, hereditary factors, metabolism, cholestasis and parasites, among other factors, can damage liver cells, destroying the dynamic balance between collagen fiber synthesis, deposition, degradation and absorption, thus leading to the development of liver fibrosis. Therefore, liver fibrosis is not a unique disease; instead, different factors can lead to it, and regardless of the factors causing the relevant liver injury, the fibrosis process is similar (Mehal et al., 2011; Sebastiani et al., 2014; Kamdem et al., 2018; Testino et al., 2018).

**FIGURE 1:**
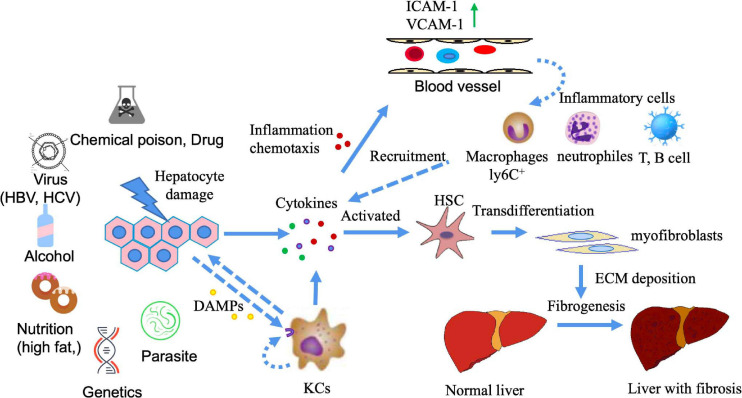
The mechanism of liver fibrosis. Liver damage can be caused by a variety of factors (e.g., chemical poisons, viruses, alcohol, nutrition, genetics, parasites, etc.). Injuries of liver cells release a variety of cytokines, among which damage-associated molecular patterns(DAMPs) (such as S100 family, high mobility group protein, etc.) can activate Kuffers (KCs) cells to produce inflammatory cytokines. Inflammatory cytokines can play a role through autocrine and paracrine. At this point, vascular permeability at the injured site increases, vascular endothelium simultaneously expresses high adhesion molecules, and inflammatory cells in the blood chemotaxis to the injured site under the action of chemokines, leading to the occurrence of inflammation. Multiple cytokines produced at the site of liver injury activate resting HSCs, causing HSCs to differentiate into myofibroblasts and produce ECM deposition. If the injury and inflammation are persistent or recurring, the deposition of ECM cannot be reversed, leading to liver fibrosis.

The stimulation of different factors can lead to liver cell damage, which in turn causes inflammation. A continuous inflammatory response often leads to the formation of fibrosis. This is because inflammation causes cell damage, which further enhances the release of inflammatory mediators, such as cytokines and chemokines. These mediators recruit a large number of inflammatory cells to the site of inflammation, such as lymphocytes, neutrophils, eosinophils, basophils, mast cells and macrophages. The collected inflammatory cells further activate effector cells, which promote fibrosis (Hernandez-Gea and Friedman, 2011; Koyama and Brenner, 2017; Parola and Pinzani, 2019). The activation of hepatic stellate cells (HSCs) is the core event of liver fibrosis. HSCs are activated and differentiated into myofibroblasts (MFBs), which secrete and deposit a large amount of extracellular matrix (ECM). When liver injury persists for a long time, chronic inflammatory stimulation and the continuous deposition of ECM together lead to the gradual replacement of normal liver tissue by fibrous tissue (Higashi et al., 2017; Zhangdi et al., 2019; Figure 1). As a common pathological stage of chronic liver disease, liver fibrosis is necessary for the development of liver cirrhosis and even liver cancer. Indeed, persistent liver inflammation and fibrosis are known to eventually induce cirrhosis and liver cancer (Uehara et al., 2013).

Studies in rodent models and humans have shown that liver fibrosis is reversible if the damage is ameliorated in a timely manner (Campana and Iredale, 2017). Matrix Metallopeptidase 13 (MMP13) is involved in the degradation of newly formed matrix during the recovery of liver fibrosis in rats. Although not all HSCs express MMP13, the production of MMP13 by HSCs plays a critical role in the process of fibrosis recovery (Watanabe et al., 2000). MMP9 secreted by Kupffer plays a key role in reversing hepatic fibrosis induced by thioacetamide (TAA) in mice (Feng et al., 2018). Dendritic cells can promote fibrosis regression by producing MMP9 (Jiao et al., 2012).

At present, the mechanism underlying liver fibrosis is unclear, and recent research has mostly focused on the etiology and mechanism of the disease. Although there has been some progress in the diagnosis and treatment of fibrosis, effective drugs and treatment are still lacking. The prevention, treatment and even reversal of liver fibrosis have always been the key to the successful treatment of chronic liver injury. The pathogenesis of liver fibrosis is of great clinical significance for the development of therapeutic drugs and overall improvement of therapeutic approaches. It is therefore important to study the pathogenesis of liver fibrosis and develop therapeutic drugs to construct the animal model of liver fibrosis with similar pathogeneses.

We herein report various liver fibrosis models, which are classified according to the modeling method used, to facilitate their utility as a reference for liver fibrosis researchers.

## Chemical Drug-Induced Liver Fibrosis Model

Chemical liver fibrosis is induced by chemicals that can cause hepatotoxicity. The liver cells are damaged and consequently repaired, resulting in the abnormal growth of connective tissue in the liver. Models of chemical-induced liver injury are usually injected intraperitoneally, which is relatively easy to perform and results in stable development for use in studies concerning clinical liver fibrosis.

### Carbon Tetrachloride (CCl_4_)

Carbon tetrachloride is a colorless non-polar organic compound, highly toxic, that can dissolve many substances such as fat and paint. It is a typical liver poison, but the concentration and frequency of exposure can affect its action site and toxicity. CCl_4_ directly damages liver cells (mainly endothelial cells and hepatic parenchyma cells in the hepatic portal vein region) by altering the permeability of lysosomes and mitochondrial membranes (Weber et al., 2003). The oxidase system in liver cells can also form highly active free radical metabolites through CYP2E1, leading to severe central lobular necrosis (Zangar et al., 2000). The damage mechanism of CCl4 is mainly oxidative damage caused by lipid peroxidation. Cytochrome P450 enzyme, especially CYP2E1, converts CCl4 into highly toxic trichloromethyl radical (⋅CCl_3_) and trichloromethyl peroxide (⋅CCl_3_O_2_) (Slater et al., 1985; Unsal et al., 2020). This model has been widely used to study the pathogenesis of liver fibrosis and cirrhosis.

More standardized procedures are needed for experimental liver fibrosis studies due to dramatic changes in animal welfare regulations in Europe. Scholten proposed standard operating procedure (SOPs) for the CCl_4_ mouse model and summarized the widely accepted experimental model for inducing liver injury leading to liver fibrosis (Scholten et al., 2015). The toxicological mechanism of liver fibrosis induced by CCl_4_ may be related to multiple biological processes, pathways and targets (Dong et al., 2016). After 15 weeks of CCl_4_ induction, multiple well-differentiated hepatocellular carcinoma (HCC) cells were found in the livers of all mice. CD133 was significantly up-regulated after CCl_4_ treatment, and the levels of desmin and glial fibrillary acidic protein, the representative markers of HSC, were also significantly increased. The EGF expression was significantly reduced, contrary to what has been observed in humans. In A/J mice, chronic liver injury induced by CCl_4_ differs from HCC induced by human cirrhosis (Fujii et al., 2010). The collagen expression was found to be significantly increased after CCl_4_ injury, and the number of cells expressing cytoglobin was also increased. Cytoglobin may be an early biomarker of liver fibrosis (Man et al., 2008). In addition to intraperitoneal injection, CCl4 can also be inhaled to establish a liver fibrosis model. Rats were exposed to CCl4 vapor twice a week for 30 s each time, while phenobarbital (0.3 g/L) was added to drinking water. The duration of inhalation was increased by 30 s after the first three sessions and by 1 min after every three sessions until a steady state was reached for 5 min. After 9 weeks, it can lead to liver fibrosis (Leeming et al., 2013; Marfà et al., 2016). Compared with intraperitoneal injection, inhalation route is a complex process, with great individual differences, and can cause multiple organ damage. An intraperitoneal injection can reach the liver directly from the hepatic portal vein. The animal model of liver fibrosis induced by CCl_4_ is relatively low-cost to develop, and the implementation method is relatively simple. Furthermore, it is a classic model and one of the earliest, most widely used and most frequently selected by researchers.

### TAA

Thioacetamide is an organic compound with the molecular formula CH_3_CSNH_2_, found as a colorless or white crystal. TAA is widely used as a model for inducing experimental liver fibrosis, and can also be used to induce acute liver failure and liver tumors by controlling the dose and duration of administration. The TAA model is suitable for the study of connective tissue metabolism in fibrotic and cirrhotic models (Müller et al., 1988). TAA itself is not hepatotoxic, and its active metabolites covalently bind to proteins and lipids, causing oxidative stress leading to central lobular necrosis of the liver. Compared with CCl_4_, TAA resulted in more periportal inflammatory cell infiltration and more pronounced ductal hyperplasia. The intraperitoneal administration of 150 mg/kg of TAA 3 times per week for 11 weeks in rats and TAA administration in drinking water at 300 mg/L for 2-4 months in mice can successfully and repetitively cause chronic liver injury and fibrosis (Wallace et al., 2015). The continued administration of TAA (after the continuous TAA injection for more than 11 weeks) induced sustained liver fibrosis in common marmosets, and this primate-like model of liver fibrosis was thus able to be used to evaluate the therapeutic effect of liver fibrosis (Inoue et al., 2018). In *Macaca fascicularis* fibrosis models induced by TAA and CCl_4_, TAA induced significant fibrosis, but CCl_4_ did not. Both TAA and CCl_4_ increased the Child-Pugh score, but only the TAA model showed an increased retention of indocyanine green. TAA-induced *M. fascicularis* fibrosis was similar to Child-Pugh grade B fibrosis in humans. This model is evaluable by clinical indicators and can be used in preclinical studies (Matsuo et al., 2020). Although both CCl_4_ and TAA-induced liver injury and fibrosis are dependent on CYP2E1, in some cases, CYP2A5 may have a protective effect against TAA-induced liver injury and fibrosis but has no effect on the hepatotoxicity of CCl_4_ (Hong et al., 2016). The serum amino acid pattern in the TAA-induced chronic cirrhosis model is partially similar to the corresponding human disease (Fontana et al., 1996). The hepatic fibrosis model of rats was established by injecting TAA solution for 7 weeks. Serum and urine samples were collected weekly for a nuclear magnetic resonance metabolomics analysis to search for differential metabolites associated with TAA-induced injury. That study helped clarify the role of metabolic dynamics in the course of hepatic fibrosis disease (Wei et al., 2014). The levels of fibrogenic cytokines, such as transforming growth factor-β(TGF-β), platelet derived growth factor (PDGF) and connective tissue growth factor (CTGF), also increased in the liver tissue of all three models, but the levels of CTGF in the liver tissue and serum were the highest in the CCl_4_ group (Park et al., 2016). After 12-week oral administration of TAA in rats, bile duct fibrosis was induced, characterized by tubular hyperplasia surrounded by fibrous tissue (Hata et al., 2013). Both CCl4 and TAA can cause lipid oxidative damage in liver cells. The model of liver fibrosis induced by CCl4 is more suitable for studying the mechanism of spontaneous reversal of liver fibrosis. The hepatic fibrosis model induced by TAA is more suitable for the study of the mechanism of hepatic fibrosis, the screening of therapeutic drugs and the reliability evaluation of hepatic fibrosis serological markers.

### DMN and Diethylnitrosamine (DEN)

The toxicity of various nitrosamines in animals and humans is well established, and trace amounts of DEN or DMN can cause severe liver injury in either the enteric or oral form. The most prominent manifestations are extensive neutrophilic infiltration, extensive central lobular hemorrhaging and necrosis, bile duct hyperplasia, fibrosis, bridging necrosis and ultimately HCC. Due to the stability of DMN- and DEN-induced liver changes, the administration of these agents to rodents has become a commonly used experimental model (Tolba et al., 2015).

Iron deposition and fat accumulation were shown to play an important role in the pathological changes of DMN-induced liver fibrosis in rats (He et al., 2007). Rats were intraperitoneally injected with DMN 3 days a week for 3 weeks. Severe central lobular congestion and hemorrhaging and necrosis were observed on day 7. On day 14, central lobular necrosis and numerous neutrophils infiltration were observed. Collagenous fibrous deposition was seen on day 21, along with severe central lobular necrosis, focal fatty changes, bile duct hyperplasia and bridging necrosis and fibrosis around the central vein. DMN-induced liver injury in rats seems to be an animal model similar to early human cirrhosis (George et al., 2001). The model shows significantly increased liver collagen fibraldehyde content due to DMN administration, and the cross-linking of liver fibrosis collagen induced by DMN is greater than that in normal liver. Furthermore, the deposition of type III collagen is more obvious than that of type I collagen in early fibrosis (George and Chandrakasan, 1996). The percentage of collagen fibrosis in rat liver fibrosis induced by DMN has been shown to be closely correlated with the serum levels of hyaluronic acid (HA), laminin (LN) and type IV collagen (Li et al., 2005). After 4 weeks of DEN treatment, 30% of zebrafish showed hyperplasia of reticular fibers. After 6 weeks, reticular and collagen fibers showed active hyperplasia, and the proliferation rate of reticular fibers increased to 80%, successfully generating a stable liver fibrosis model in zebrafish (Wang et al., 2014).

In the comparative study of dimethylnitrosamine (DMN), CCl4 and TAA rat liver fibrosis models, lipid peroxidation was highest in the CCl4 model, and the serum liver enzyme levels increased with severity. The DMN and TAA models showed significant changes in liver fibrosis. The Alpha-SAM levels significantly increased in the DMN model. In summary, while the modeling time with this method is short, its development is simple, and the fibrosis degree is stable. However, because of the toxicity of nitrosamines, researchers should ensure proper safety measures are taken.

### Acetaminophen (APAP)

Acetaminophen overdose is a major cause of drug-induced acute liver failure in many developed countries. Mitochondrial oxidative stress is considered the core event of APAP-induced liver injury (Yoon et al., 2016; Yan et al., 2018). N-acetyl-p-phenylquinone imine (NAPQI), a metabolite of APAP, is hepatotoxic and can increase the mRNA expression of α-SMA, COL1A1, COL3A1 and TGF-β, inducing the phosphorylation of ERK1/2 and SMAD2/3 and nuclear translocalization of EGR-1 in hepatic stellate LX2 cells. The long-term administration of APAP can induce liver fibrosis in mice (Bai et al., 2017). When the liver is first exposed to APAP, a necrotizing inflammatory process is kicked off, followed by liver regeneration. However, the liver begins to form fibrosis after the second exposure to APAP (AlWahsh et al., 2019). Of note, a new model of cirrhosis in which rats were gavaged with corn oil daily and APAP 500 mg/day for 3 weeks resulted in the development of focal biliary cirrhosis (Tropskaya et al., 2020).

## Immune Damage-Induced Liver Fibrosis Model

Immune liver injury liver fibrosis mainly refers to liver fibrosis caused by clinical autoimmune hepatitis (AIH) and virus infection, but such animal models of immune liver injury lack the sustained replication stage of human virus infection and the pathological process of sustained damage of human liver immunity.

Concanavalin A (ConA), a lectin purified from Brazilian kidney bean (Soares et al., 2011), is widely used in the mouse model of immune-mediated hepatitis. Unlike other models of liver injury, ConA-induced injury is mainly caused by the activation and recruitment of T cells to the liver (Heymann et al., 2015). Therefore, the pathogenesis of the ConA model has something in common with human immune-mediated hepatitis, such as AIH (Wang et al., 2012) and viral hepatitis. The mouse hepatitis model induced by ConA (20 mg/kg, 12 h) reflects most of the pathogenicity of human type I AIH. This provides a reliable animal model for the study of the immune pathogenesis of AIH and the rapid evaluation of new therapeutic methods (Ye et al., 2018). In acute autoimmune liver injury induced by ConA, HSCs are activated early, and the expression of TGF-β1 and TGF-β3 is unbalanced, which may be related to liver dysfunction and fibrosis development (Wang et al., 2017). Repeated injections of Con A resulted in liver fibrosis in mice (Louis et al., 2000). The model of immune fibrosis in mice was established by injecting saffra protein A (0.3 mg/body) once a week for 4 weeks. IFN-β can inhibit liver cell damage caused by repeated injections of ConA but has no effect on the development of fibrosis (Tanabe et al., 2007).

## Alcohol-Induced Liver Fibrosis Model

Alcoholic liver disease (ALD) is a chronic liver disease caused by long-term heavy drinking. Fatty liver is usually present in the initial stage, which can develop into alcoholic hepatitis, alcoholic liver fibrosis and alcoholic cirrhosis. Almost all heavy drinkers develop fatty liver, but only 20-40% develop more severe ALD, and the underlying mechanism leading to disease progression is still unclear (Tsukamoto and Lu, 2001; Seitz et al., 2018). Although rodents differ from humans with regard to their alcohol metabolism (Holmes et al., 1986) and immune system, experimental animal models of ALD, especially rodent models, have been widely used in the study of human ALD (Mathews et al., 2014; Lamas-Paz et al., 2018).

After the daily administration of alcohol to rats for 16 weeks, the rates of liver steatosis, necrosis, inflammation and fibrosis were increased (Zhou et al., 2013). Chronic ethanol feeding (10 days free oral Lieber-decarli ethanol liquid diet) plus single alcoholic ethanol feeding induced liver injury, inflammation and fatty liver, simulating acute and chronic alcoholic liver injury in humans. This simple model is very useful for the study of ALD and other organs damaged by alcohol consumption (Bertola et al., 2013). Mice treated with CCl_4_ combined with ethanol (up to 16%) showed extremely high rates of fibrotic alcoholic fatty liver disease 7 weeks later. The pattern of steatosis, inflammation and fibrosis involved in ALD in this mouse model is similar to that in humans and is suitable as a preclinical model for drug development (Brol et al., 2019). The same CCl_4_ vapor exposure combined with chronic alcohol feeding resulted in extensive liver fibrosis in rats at week 5 and micronodular cirrhosis at week 10. This animal model simulates how some chronic liver damage in humans may be due to the presence of other hepatotoxins in the environment that play a role in enhancing the effects of alcohol (Hall et al., 1991). A new experimental model of porcine hepatosclerosis was established by CCl_4_ and ethanol. Cirrhosis was induced by the intraperitoneal injection of CCl_4_ twice a week for 9 weeks. Corn flour was the only food consumed during the period, and a 5% alcohol-water mixture was consumed. After 9 weeks, 83.3% of the pigs had cirrhosis, and 33.3% had died (Zhang et al., 2009). In combination with chronic alcohol administration and a non-alcoholic steatohepatitis (NASH)-induced high-fat diet, this new model enables the study of the combined effects of alcohol and a high-fat diet on liver injury, which may contribute to the development of liver fibrosis by enhancing TLR_4_ signaling (Gäbele et al., 2011). To cause progressive alcoholic liver injury, the animal must be given too much alcohol and maintain a persistently high blood alcohol level. Because of the rats’ natural aversion to alcohol, the method of feeding them alcoholic liquid food was greatly restricted. In addition, the experiment cycle is long, the cost is high and the success rate is low, so it has been rarely used. At present, the more commonly used method is alcohol combined with chemical poison gavage, during the control of diet to replicate the model of alcoholic liver fibrosis. The model has the advantages of simple operation, short cycle and high molding rate.

## Diet Metabolism-Induced Liver Fibrosis Model

Non-alcoholic fatty liver disease (NAFLD) is a clinicopathological syndrome characterized by excessive fat deposition in hepatocytes except for alcohol and other clear liver damage factors, which is closely related to insulin resistance and genetic susceptibility of acquired metabolic stress liver injury (Cobbina and Akhlaghi, 2017). NAFLD is becoming a common chronic liver injury due to lifestyle changes. NAFLD can cause inflammation, ballooning degeneration of hepatocytes, and varying degrees of fibrosis, known as non-alcoholic steatohepatitis (NASH). Patients with advanced liver fibrosis or cirrhosis are at risk of developing complications, such as HCC and esophageal varices (Stål, 2015).

A choline-deficient high-fat (CDHF) diet induces NASH in mice. Hepatic histopathology has shown that a CDHF diet causes severe steatosis, inflammation and pericellular fibrosis (Honda et al., 2017). A modified choline-deficient, L-amino acid-defined, high-fat diet (CDAHFD) rapidly induces liver fibrosis in mice. This model will contribute to a better understanding of human NASH disease and may be useful for the development of effective treatments (Matsumoto et al., 2013). AIM^–/–^ mice fed the D09100301 diet showed similar phenotypes to non-obese patients with NAFLD, indicating their utility as a pathophysiological model for studying obesity-induced HCC (Komatsu et al., 2019). Dietary control combined with chemical toxicants may be an effective means of reducing the modeling time of diet-induced NAFLD models. A fast food diet (FFD) combined with a trace dose of CCL_4_ (0.5 mL/kg body weight) for 8 weeks resulted in histological features of NAFLD, including steatosis, inflammation and fibrosis, in Wistar rat models within 8 weeks, suggesting that the model has potential utility in developing NAFLD and anti-fibrosis therapy (Chheda et al., 2014). Furthermore, using a high-fat, high-fructose and high-cholesterol diet combined with a weekly low dose of CCL_4_ as an accelerant shortened the cycle of a mouse NASH model of fibrosis and HCC (Tsuchida et al., 2018). The mechanism of fatty liver fibrosis is still unclear, among which oxidative stress/lipid peroxidation is an important cause of fatty liver fibrosis. Unlike alcoholic fatty liver, which leads directly to liver fibrosis, non-alcoholic fatty liver must pass through an intermediate stage of steatohepatitis before it can develop into liver fibrosis. In other words, inflammation itself is a prerequisite for fatty liver fibrosis. The diet-induced NASH mouse model is characterized by good simulation of obesity, type 2 diabetes mellitus, dyslipidemia, and metabolic syndrome, but with less liver fibrosis.

## Surgery-Induced Liver Fibrosis Model

Cholestasis is an obstruction of both bile flow formation and excretion. Continuous cholestasis leads to chronic inflammation, which damages bile duct cells and liver cells, activates MFBs through a number of regulatory factors and causes the excessive deposition of ECM, leading to liver fibrosis (Li and Apte, 2015).

Surgical bile duct ligation (BDL) is one of the most widely used experimental models of cholestatic liver injury in mice and rats. The BDL model is a classic model of liver fibrosis. BDL was first achieved by the double ligation of bile ducts in rats. In brief, at 7-10 days after surgery, bile duct stenosis, increased bile duct pressure, upstream dilatation of bile ducts, increased liver volume composed of portal vein and bile duct hyperplasia are observed (Rodríguez-Garay et al., 1996). BDL protocols have been improved over time, but basically, animals are anesthetized and then undergo laparotomy. The bile duct is exposed from the abdominal cavity and ligated twice using a surgical cord. Mice and rats that undergo the procedure develop a strong fibrotic response (Kirkland et al., 2010). During the procedure, the animal can be placed on a heated plate at 37°C and permanently connected to the anesthesia system. At the time of the operation, the bile ducts are double-ligated but not dissected. This procedure induces highly repeatable morphological phenotypic changes in the liver and allows for the study of fiber formation at specific points in time (Tag et al., 2015). The standard model for cholestasis studies is total BDL (tBDL), but this model can cause severe liver damage in mice, so a new cholestasis model using partial BDL (pBDL) has been established (Heinrich et al., 2011). A mouse model of recanalization of biliary tract obstruction was previous established by performing anastomosis between the gallbladder and jejunum (G-J anastomosis), which has some value for studying the recovery from cholestasis liver fibrosis (Yoshino et al., 2021).

## Transgenic Animal Liver Fibrosis Model

A chronic hepatitis C virus infection leads to liver fibrosis and cirrhosis. For a long time, chimpanzees were the only available non-human model of HCV infection (Pellicoro et al., 2014). Since the host range of HBV is relatively narrow and it only infects humans, it is very difficult to establish an animal model of HBV infection. Only chimpanzees and tupaia have previously been used for infection experiments (Walter et al., 1996; Dandri et al., 2005). The construction of humanized liver chimeric transgenic mice enables the stable regrowth of human liver cells in mice, and even the normal function and morphology of human liver, which has become an important bridge between mouse and human preclinical studies (Katoh et al., 2008). Hepatitis virus infects human TK-NOG mice and UPA-SCID mice with severe combined immunodeficiency (UPA-SCID). All human TK-NOG and UPA-SCID mice injected with hepatitis B virus infected serum developed viremia. The occurrence of HCV viremia in TK-NOG mice was significantly higher than that in UPA-SCID mice. TK-NOG mice are more beneficial for the study of hepatitis virus virology and the evaluation of antiviral drugs (Kosaka et al., 2013).

In addition to humanized mice, many transgenic mice were constructed for the study of liver fibrosis according to the different pathogenesis of liver fibrosis and the key functional genes of liver fibrosis regulation (Hayashi and Sakai, 2011). Immunodeficiency NOD induced natural killer T cell (NKT) transgenic population mediated spontaneous multi-organ chronic inflammation and fibrosis, non-obese diabetic inflammation and fibrosis (N-IF) mice. Due to fibrosis components, early onset, spontaneity, and reproducibility, this novel mouse model provides further insight into the underlying mechanisms that mediate the transformation of chronic inflammation into fibrosis (Fransén-Pettersson et al., 2016). Although the pathology of BDL is similar to chronic cholestasis in humans, the severity of surgical stress and cholestasis injury limits the application of the BDL model. MDR2 (ABCB4) is a mouse homologous gene MDR3 (ABCB4) that encodes a tubule phospholipid transporter. MDR2-/- mice, also known as ABCB4-/- mice, are another mature model of chronic cholestatic liver injury (Ikenaga et al., 2015). MDR2 knockout (MDR2 -/-) mice are a genetic model similar to patients with primary sclerosing cholangitis (Nishio et al., 2019). Transgenic mice that overexpress the transforming growth factor-β1 (TGF-β1) fusion gene [C-reactive protein (CRP)/TGF-β1] are able to control the expression level of TGF-β1. This model can be used to study the regulation of collagen synthesis, fibrinolysis and the degree of reversibility of liver fibrosis. CRP/TGF-β1 transgenic mouse model can be used as an anti-fibrofactor test model (Kanzler et al., 1999). Similarly, TGF-β1 overexpression transgenic mice were established based on the tetracycline regulation gene expression system. This model will help to analyze the role of TGF-β1 in fibrogenesis (Ueberham et al., 2003). The role of platelet-derived growth factor A (PDGF-A) in the formation of liver fibrosis *in vivo* can be evaluated in transgenic mice with hepatocellular specific overexpression of PDGF-A by the C-reactive protein (CRP) gene promoter (Thieringer et al., 2008). Metalloproteinase-1 tissue inhibitor (TIMP-1) is upregulated during liver fibrogenesis, but its role in liver fibrosis and carcinogenesis in mice is not necessarily direct (Thiele et al., 2017). Transgenic mice overexpressing human TIMP-1 (HTIMP-1) in the liver under the control of albumin promoter/enhancer can be used to investigate the role of TIMP-1 in promoting liver fibrosis (Yoshiji et al., 2000). Mouse models carrying human apolipoprotein E^∗^ 3-leiden and cholesterol ester transfer protein, fed a “Western” diet, lead to liver inflammation and fibrosis that are highly dependent on genetic background and have a large overlap of pathways between human diseases (Hui et al., 2018).

## Organoid Liver Fibrosis Modes

Studying tissue and organ biology in mammals is challenging, and progress may be hampered by the availability of samples and ethical issues, especially in humans (Rossi et al., 2018). Although traditional 2D cell culture systems have many advantages, these models lack the ability to maintain *in situ* cellular characteristics and reflect cell-to-cell and cell-to-matrix interactions. The primary cells obtained by purification and isolation will also lose their original functions and characteristics after 2D culture *in vitro*. Organoids are 3D organ-like cells that are derived from embryonic or adult stem cells that are cultured *in vitro* and have a definite structure and function. Although these cellular structures are not human organs in the true sense, they can mimic real organs in structure and function, so they are playing an increasingly important role in scientific research. Organoids *in vitro* culture systems are characterized by self-renewing stem cell populations that include cells capable of differentiating into organs with similar spatial tissue functions (Artegiani and Clevers, 2018). Organoids can be used to simulate organ development and disease, and have a wide range of applications in basic research, drug development, and regenerative medicine (Lancaster and Knoblich, 2014; Huch et al., 2017; Xia et al., 2019). While mouse models and cell lines have advanced our understanding of liver biology and related diseases, they have significant drawbacks in simulating human liver tissue, particularly its complex structure and metabolic function. Currently, a variety of liver organoids have been established from induced pluripotent stem cells, embryonic stem cells, hepatoblasts and adult tissue-derived cells (Prior et al., 2019).

HepaRG (Hep) and primary human HSCs were cultured into 3D spheres in 96-well plates. The metabolic capacity of the organoid exceeds 21 days. This novel liver organ culture model is the first capable of detecting hepatocellular dependence and compound-induced HSC activation and represents an important advance in the *in vitro* compound assessment of drug-induced liver fibrosis (Leite et al., 2016). Induced pluripotent stem cell-hepatic stellate cells (iPSC-HSCs) are very similar to primary human HSCs at the transcriptional, cellular, and functional levels. iPSC-HSCs exhibit a static phenotype when they remain 3D spherical with HepaRG hepatocytes, but are activated in response to wound-healing mediator stimulation and hepatocytotoxicity, resulting in fibrotic responses and secretion of procollagen, and accumulation of retinol in lipid droplets, similar to their *in vivo* counterparts. Thus, this protocol provides a powerful *in vitro* system for studying stellate cell development, modeling liver fibrosis, and screening for drug toxicity (Coll et al., 2018). Activated hepatic stellate cells (aHSCs) produced by 2D culture were coated in a 3D collagen gel to form a spherical structure, which created a stiffer environment and expressed higher levels of TIMP1 and LOXL2 compared to LX-2 cells cultured in 2D culture. This model proposes a fibrosis model that can be combined with the multicellular model to more accurately reflect the impact of severe fibrosis on liver function (Brovold et al., 2020). Using organoids from intrahepatic bile ducts, APAP was used to induce organoid injury in culture medium. The injury model suggested that bile duct cell apoptosis and its fibrotic response played a role in the initiation of the fibrotic process of bile duct diseases, such as biliary atresia (BA) (Chusilp et al., 2020). Genetically susceptible NAFLD organoid systems composed of hepatocytes (HepG2) and HSCs (LX-2) can be used to clarify the molecular mechanisms underlying the accumulation of lipids that induces the early stage of fibrogenesis. In addition, these systems can be used to identify new compounds for treating NASH through high-throughput drug screening (Pingitore et al., 2019). It is difficult to select media and extracellular matrix that can co-maintain multiple cell lineages. A novel organ-like culture method was developed for co-differentiation of epithelial and mesenchymal lineages from PSCs. Using 11 different health and disease pluripotent cell lines, a repeatable method was developed to obtain multicellular human liver-like organs composed of hepatocytes, stellate cells, and Kupffer like cells that exhibit transcriptome similarity to tissue of *in vivo* origin. These multicellular human hepatic organs (HLOs), in combination with free fatty acid therapy, reproduce the progressive, staging nature of steatohepatitis like pathology, including steatosis, inflammation, and fibrosis, and can potentially be used for drug screening by analyzing organ hardness (Ouchi et al., 2019). Liver organoids were generated from mice with mild (NASH A), moderate (NASH B), and severe (NASH C) methionine and choline deficiency diets-induced NASH models that reproduce the characteristics of NASH disease liver tissue. The NASH liver organoid model can be used to study genetic stability and/or lipid metabolism during NAFLD/NASH transformation (Elbadawy et al., 2020).

**FIGURE 2:**
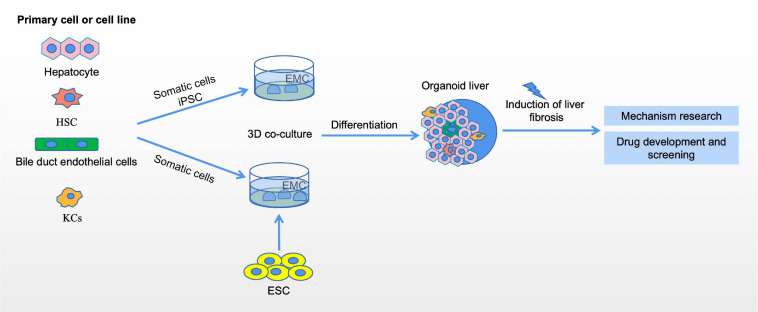
Organoid models of liver fibrosis. The generation of liver organoids and liver fibrosis models. liver organoid can be constructed by 3D co-culture of embryonic stem cells (ESC) and somatic hepatocytes, 3D coculture of induced pluripotent stem cells, and then through induction and differentiation of different developmental stages, and finally can be developed into liver organoids in the reactor. The establishment of liver fibrosis model using liver organoids can be used to study the mechanism of liver fibrosis and drug development and screening.

In conclusion, organoid technology is one of the most important advances in stem cell research. Organoids are three-dimensional cell cultures that reproduce some of the key cell types and structural characteristics of the organs they represent. Organoids remove the confounding variables that might be introduced by animal models and are more complex than homogenized cell cultures. Organoid culture has a high degree of gene stability, maintaining the genotype and phenotype of the source tissue. Thus, organoids can be used to model diseases, to study the mechanisms and progression of diseases, and to predict patients’ individual responses to drug therapy (Figure 2).

## Conclusion and Perspectives

**FIGURE 3:**
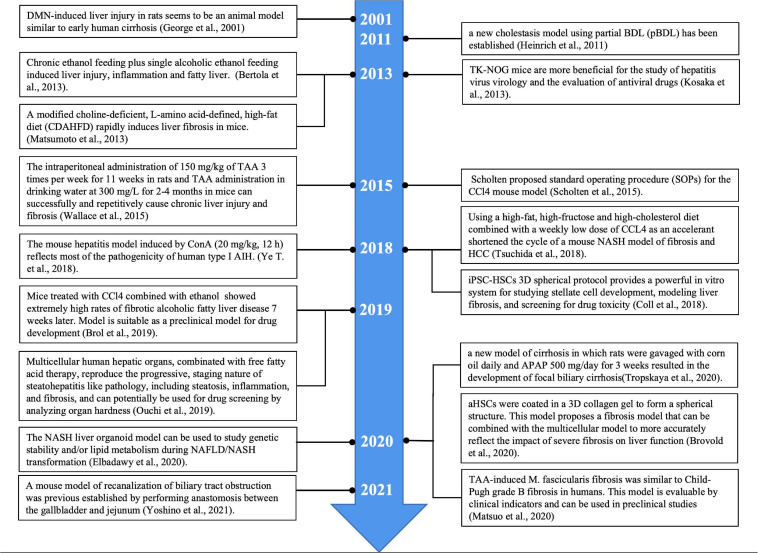
Timeline of animal and organoid liver fibrosis mode.

A reasonable model of liver fibrosis should resemble the characteristics and pathogenesis of human disease. It is universally recognized that “animal welfare” includes five freedoms: freedom from hunger and thirst; comfort; freedom from pain, injury and disease; freedom from fear and sadness and expression of nature. In laboratory animals, it is difficult to achieve all five freedoms at the same time. In particular, damage to these creatures’ health is often a byproduct of the natural course of research. Due to such factors as animal suffering and scientific exploration, the three R principles are widely recognized: including replacement, reduction and refinement (Lindsjö et al., 2016). This requires animal experiments to have stable experimental methods, a high mold-forming rate and good reproducibility. Thus far, researchers have successfully developed a number of hepatic fibrosis models using different experimental animals and different methods. However, due to the complexity of the pathogenesis of human liver fibrosis and differences in the genetic background between human and other animal species, there is no modeling method that can perfectly replicate the process of human liver fibrosis. Researchers can only use different models of liver fibrosis to mimic, as much as possible, the different pathologies that cause liver fibrosis in humans. Table 1 briefly compares the advantages and disadvantages of different liver fibrosis models (Table 1). Although the liver fibrosis model induced by chemical poisons is different from the pathogenesis of human liver fibrosis, it is often used to study the mechanism of liver fibrosis due to its simple operation and good reproducibility. The immune-induced liver injury fibrosis model most closely replicates the clinical situation, which is similar to the liver fibrosis caused by human AIH and virus infection. Due to the complex pathogenesis of ALD and NAFLD, and the great differences between animal genetics, metabolism and immunity and human beings, it is relatively difficult to construct a model similar to human diseases through liver fibrosis induced by alcohol and the diet metabolism. BDL can simulate liver fibrosis induced by cholestasis, requiring only a short time with good reproducibility for model construction. However, it has drawbacks of substantial operational requirements, the need for an aseptic surgical setting and high animal mortality. Humanized transgenic chimeric mice and transgenic/knockout mice are emerging modeling methods that have been established in recent decades. Humanized liver chimeric transgenic mice constitute a good animal model of hepatitis virus infection, and transgenic/knockout mice are a good animal model for studying the role of the functional genes involved in liver fibrosis. The latest organoid model makes up for the great difference between the traditional *in vitro* cell culture and human organs. Liver organoids are expected to be a useful new model for *in vitro* experiments that closely resembles the actual situation in human liver diseases. Although the techniques are becoming more advanced, liver fibrosis models are becoming more complex. Researchers continue to make various models of liver fibrosis in order to bring them closer to the true pathogenesis of human liver fibrosis (Figure 3). Each model has its advantages and disadvantages, and it remains a challenge to identify the most reasonable and stable hepatic fibrosis model.

**TABLE 1:** Advantages and disadvantages of different methods in hepatic fibrosis models.

Model type	Model	species	Method	Advantages	Disadvantages	References
Chemical drug-induced liver fibrosis model	Carbon tetrachloride	Mouse/Rat	Intraperitoneal injection/inhalation	Simplicity of operator, commonly used, high reproducibility	Highly toxic and volatile, different from human liver fibrosis	Slater et al., 1985; Weber et al., 2003; Leeming et al., 2013; Scholten et al., 2015; Marfà et al., 2016; Unsal et al., 2020
	Thioacetamide	Mouse/Rat/Monkey	Intraperitoneal injection	Simplicity of operator, commonly used, high reproducibility	Highly toxic	Müller et al., 1988; Fontana et al., 1996; Wei et al., 2014; Wallace et al., 2015; Inoue et al., 2018
	Dimethylnitrosamine or diethylnitrosamine	Mouse/Rat/Zebra fish	Intraperitoneal injection	Simplicity of operator, commonly used, high reproducibility	Highly toxic and volatile	George et al., 2001; Wang et al., 2014; Tolba et al., 2015
	Acetaminophen	Mouse/Rat	Intraperitoneal injection, gavage	Simplicity of operator, similar to the drug liver fibrosis	—	Yoon et al., 2016; Yan et al., 2018; Tropskaya et al., 2020
Immune damage-induced liver fibrosis model	Concanavalin A	Mouse/Rat	tail vein injection	High success rate, low animal mortality and simple operation	Similar to liver fibrosis caused by chronic virus or autoimmunity in humans	Tanabe et al., 2007; Wang et al., 2012; Heymann et al., 2015; Ye et al., 2018
Alcohol-induced liver fibrosis model	Alcohol	Mouse/Rat	Gavage	Suitable for the study of alcoholic liver disease	Alcohol tolerance in rodents	Bertola et al., 2013; Zhou et al., 2013
	Alcohol combined with chemical poisons	Mouse/Rat/Pig	Gavage	Suitable for the study of alcoholic liver disease, short model cycle	Alcohol tolerance in rodents	Hall et al., 1991; Zhang et al., 2009; Brol et al., 2019
Diet metabolism-induced liver fibrosis model	Dietary deficiencies	Mouse/Rat	Feeding	Close to human NASH	Long time t develop mild fibrosis	Matsumoto et al., 2013; Honda et al., 2017
	Dietary deficiencies combined with chemical poisons	Mouse/Rat	Feeding	Close to human NASH	—	Chheda et al., 2014; Tsuchida et al., 2018
Surgery-induced liver fibrosis model	Surgical bile duct ligation	Mouse/Rat	Surgery	Fast molding and high repeatability Close to human cholestatic injury	Surgical operation required, high mortality rate	Rodríguez-Garay et al., 1996; Kirkland et al., 2010; Heinrich et al., 2011; Tag et al., 2015
Transgenic animal liver fibrosis model	Humanized liver chimeric transgenic mice	Mouse	Transgenic mice combined with human hepatocyte transplantation	Simulate the process of human infection with hepatitis virus	Transplant surgery is complicated. The source of primary human hepatocytes was deficient	Katoh et al., 2008; Kosaka et al., 2013
	Transgenic/knockout mice	Mouse	Transgenic mice	Identify the role of a gene in liver fibrosis	Long time to develop Expensive price	Kanzler et al., 1999; Ueberham et al., 2003; Thieringer et al., 2008; Fransén-Pettersson et al., 2016; Nishio et al., 2019; Yoshiji et al., 2000
Organoid liver fibrosis modes	Liver organoids	Human/Mouse	3D *in vitro* culture technology	Homology with target organs Functionally similar with target organs	High model cost Difficulty of model Uncontrollable factors	Leite et al., 2016; Artegiani and Clevers, 2018; Coll et al., 2018; Ouchi et al., 2019; Pingitore et al., 2019; Prior et al., 2019; Brovold et al., 2020; Chusilp et al., 2020; Elbadawy et al., 2020

## Author Contributions

All authors contributed to the writing and editing of the manuscript and contributed to the article and approved the submitted version.

## Conflict of Interest

The authors declare that the research was conducted in the absence of any commercial or financial relationships that could be construed as a potential conflict of interest.
